# Supplementary material for: A BRET biosensor for measuring uncompetitive engagement of PRMT5 complexes in cells
Source: Nat Commun. 2025 Dec 3;16:10129. doi: 10.1038/s41467-025-65558-6 (PMC12675575; doi:10.1038/s41467-025-65558-6)

A BRET Biosensor for Measuring Uncompetitive Engagement of PRMT5 Complexes in Cells

Elisabeth M. Rothweiler[1,2‡], Ani Michaud[3‡], Jakub Stefaniak[1,2], Usha Singh[1,2], Brynwood B. Mikulsky[3], James D. Vasta[3], Michael T. Beck[3], Jennifer Wilkinson[3], Jennifer A. Ward[1,2], Catherine M. Rogers[1,2], Esra Balıkçı[1,2], Jeppe Tranberg-Jensen[1,2], Jesper S. Hansen[1,2], Peter Loppnau[4], Adrian Whitty[5], Paul E. Brennan[1,2,6], Peter J. Tonge[7,8*], Matthew B. Robers[3*], and Kilian V. M. Huber[1,2*]

[1]*Centre for Medicines Discovery, Nuffield Department of Medicine, University of Oxford, Old Road Campus, Roosevelt Drive, Oxford OX3 7FZ, UK*
[2]*Target Discovery Institute, Nuffield Department of Medicine, University of Oxford, Old Road Campus, Roosevelt Drive, Oxford OX3 7FZ, UK*
[3]*Promega Corporation, Madison, WI, USA*
[4]*Structural Genomics Consortium, University of Toronto, 101 College St, Toronto, ON M5G 1L7, Canada*
[5] *Department of Chemistry, Boston University, Boston, MA, 02215 USA*
[6]*Alzheimer's Research UK Oxford Drug Discovery Institute, Nuffield Department of Medicine, University of Oxford, Old Road Campus, Roosevelt Drive, Oxford OX3 7FZ, UK*
[7]*Center for Advanced Study of Drug Action, Department of Chemistry, Stony Brook University, Stony Brook, NY, 11794 USA*
[8]*Department of Biomedical Genetics, University of Rochester, Rochester NY 14642 USA*

Supplementary Information

1. **Supplementary** Methods **(S2-S10)**
2. **Supplementary Figures, Tables, and References (S11-S28)**
3. **Abbreviations**

   PRMT5, Protein Arginine Methyl Transferase 5; MTA, methylthioadenosine, MAT2A; methylthioadenosyltransferase 2a, SAM; S-adenosylmethionine; TE, target engagement; BRET, bioluminescence energy transfer, MTAP, S-methyl-5'-thioadenosine phosphorylase;

Supplementary Methods

1. Synthetic Procedures

All solvents were purchased as HPLC grade from commercial suppliers (Sigma, Thermo Fisher) and used without further purification. Anhydrous solvents were purchased from Acros Organics and stored under a nitrogen atmosphere with activated molecular sieves. CBH-001 was purchased from Enamine. NanoBRET 590 SE, NanoBRET™ Nano-Glo® Detection System and CellTiter-Glo® Luminescent Cell Viability Assay was purchased from Promega.

Reaction progress was monitored by TLC (Thin Layer Chromatography) and LC-MS. For the silica gel TLC, aluminium plates coated with 0.25 mm $60F_{254}$ silica gel (Merck) were used and visualized with UV light at l = 254 nm or l = 365 nm. 1H NMR spectra were recorded using a Bruker Avance 400 MHz spectrometer (400 MHz) and the deuterated solvent stated. Chemical shifts (δ) are quoted in parts per million (ppm) and referenced to the residual solvent peak. Multiplicities are denoted as singlet (s), doublet (d), triplet (t), quartet (q) and quintet (p) and derivatives thereof and multiplets (m). (br) denotes a broad resonance peak. Coupling constants are recorded as Hz and rounded to the nearest 0.1 Hz. Spectra were analysed using MestreNova 14.2.1.

LC-MS chromatograms determining mass and purity were obtained using a Waters Auto purification System equipped with Waters 2489 UV/Vis or Water 2998 Photodiode Array detectors, Waters 2424 ELS detector and SQ Detector 2 or Acquity QDa mass detector. A Phenomenex Kinetex 5 µm EVO C18 100A, 3 x 100 mm column was used for analytical measurements and a Phenomenex 5 µm EVO C18 100 A, 21.2 x 150 mm column was used for preparative HPLC purifications using a gradient program (eluent I: acetonitrile/water = 5/95 with 20 mM ammonium acetate buffer, pH 6.0; eluent II: acetonitrile/water = 80/20 with 20 mM ammonium acetate buffer, pH 6.0). Compound names were generated using ChemBioDraw Ultra v19.0 systematic naming.

(*S*)-6-((1-(5-aminopentanoyl)piperidin-4-yl)amino)-N-(3-(3,4-dihydroisoquinolin-2(1H)-yl)-2-hydroxypropyl)pyrimidine-4-carboxamide (CBH-001)

Step A

5-(((tert-butoxy)carbonyl)amino)pentanoic acid (1) (2.16 g; 9.94 mmol), benzyl N-(piperidin-4- yl)carbamate hydrochloride (2) (2.96 g; 10.9 mmol), DIPEA (2.25 mL; 23.5 mmol) and 1-hydroxy-7-azabenzotriazole (2.03 g; 14.9 mmol) were mixed in dichloromethane (50 mL). Then EDCI (basic form) (2.01 g; 12.9 mmol) was added dropwise at cooling (ice bath). The reaction mixture was left stirring at RT for 12 h. Then it was washed with NaCl 5% aqueous solution (2 × 50 mL), citric 15% aqueous solution (50 mL), NaCl 5% aqueous solution (2 × 50 mL). Then it was dried over Na_2SO_4, filtered, and evaporated to give compound 3 (3.86 g, 8.91 mmol, 89.6% yield).

Step G

To a solution of compound 3 (3.86 g; 8.91 mmol) in MeOH (150 mL) 10% Pd/C (0.948 g, 10 mol%) was added. The reaction mixture was connected to the source of hydrogen and was left stirring for 24 h. Then precipitate was filtered off; washed with MeOH (5 × 20 mL), the filtrate was evaporated, and dried in vacuo to give compound 4 (2.67 g, 11.6 mmol, 100% yield).

Step H

To a solution of (*S*)-6-chloro-N-(3-(3,4-dihydroisoquinolin-2(1H)-yl)-2-hydroxypropyl)pyrimi-dine-4-carboxamide (compound 5, 0.621 g; 1.79 mmol) and amine 4 (0.804 g; 2.69 mmol) in DMA (8 mL), DIPEA (0.63 mL; 3.58 mmol) was added. The reaction mixture was heated to 110°C (oil bath) and was left stirring for 24 h. Then it was cooled to RT, diluted with $NaHCO_3$ aqueous saturated solution (40 mL), and extracted with dichloromethane (3 × 50 mL). The combined organic layers were washed with 5% aqueous solution of NaCl (5 × 100 mL), brine (100 mL), dried over Na_2SO_4, filtered, and evaporated under reduced pressure. The crude product was purified by column chromatography ($CHCl_3$:MeOH 24:1, eluent $CHCl_3$:MeOH gradient from 24:1 to 14:1, (TLC $CHCl_3$:MeOH 9:1 Rf = 0.5) to give compound 6 (0.847 g, 1.39 mmol, 77.6% yield).

Step I

To a solution of compound 6 (0.85 g; 1.39 mmol) in dichloromethane (80 mL) TFA (20 mL, 261 mmol) was added. The reaction mixture was left stirring at RT for 24 h. Then solvent was

removed in vacuo. The crude was diluted with NaCl 5% aqueous solution (50 mL), 25% aqueous solution NH_3 (10 mL), extracted with dichloromethane (5 × 100 mL). Combined organic layer was washed with brine (100 mL), dried over Na_2SO_4, filtered; evaporated, and dried in vacuo to give pure compound CBH-002 (0.58 g, 1.14 mmol, 82 % yield).

Purity was determined by LC/MS as 97.2%. LC/MS (ESI+1) found 510.34 g/mol (509.31 g/mol calculated for $C_{27}H_{39}N_7O_3$). HR-ESI-MS $[M + H]^+$ found 510.3198 g/mol (calculated 510.3192 g/mol). ^1H NMR: (400 MHz, DMSO) δ 8.75 (t, J = 5.9, 5.9 Hz, 1H), 8.29 (s, 1H), 7.80 (d, J = 5.9 Hz, 1H), 7.08 (m, 4H), 7.01 (m, 1H), 4.96 (br s, 1H), 4.24 (d, J = 12.0 Hz, 1H), 4.12 (m, 1H), 3.86 (m, 2H), 3.59 (m, 2H), 3.44 (m, 2H), 3.31 (m, 2H), 3.15 (m, 2H), 2.82 (m, 2H), 2.72 (m, 4H), 2.30 (t, J = 7.3, 7.3 Hz, 2H), 1.91 (m, 2H), 1.68 (m, 2H), 1.50 (p, J = 7.9, 7.9, 7.7, 7.7 Hz, 2H), 1.34 (p, J = 7.5, 7.5, 7.3, 7.3 Hz, 3H), 1.23 (m, 1H). ^{13}C NMR: (126 MHz, DMSO) δ 170.49, 163.00, 157.51, 134.96, 134.19, 128.33, 126.33, 125.86, 125.36, 103.48, 79.20, 66.37, 62.65, 56.16, 51.23, 46.96, 44.24, 43.63, 41.44, 33.00, 32.24, 31.84, 31.02, 28.68, 22.38.

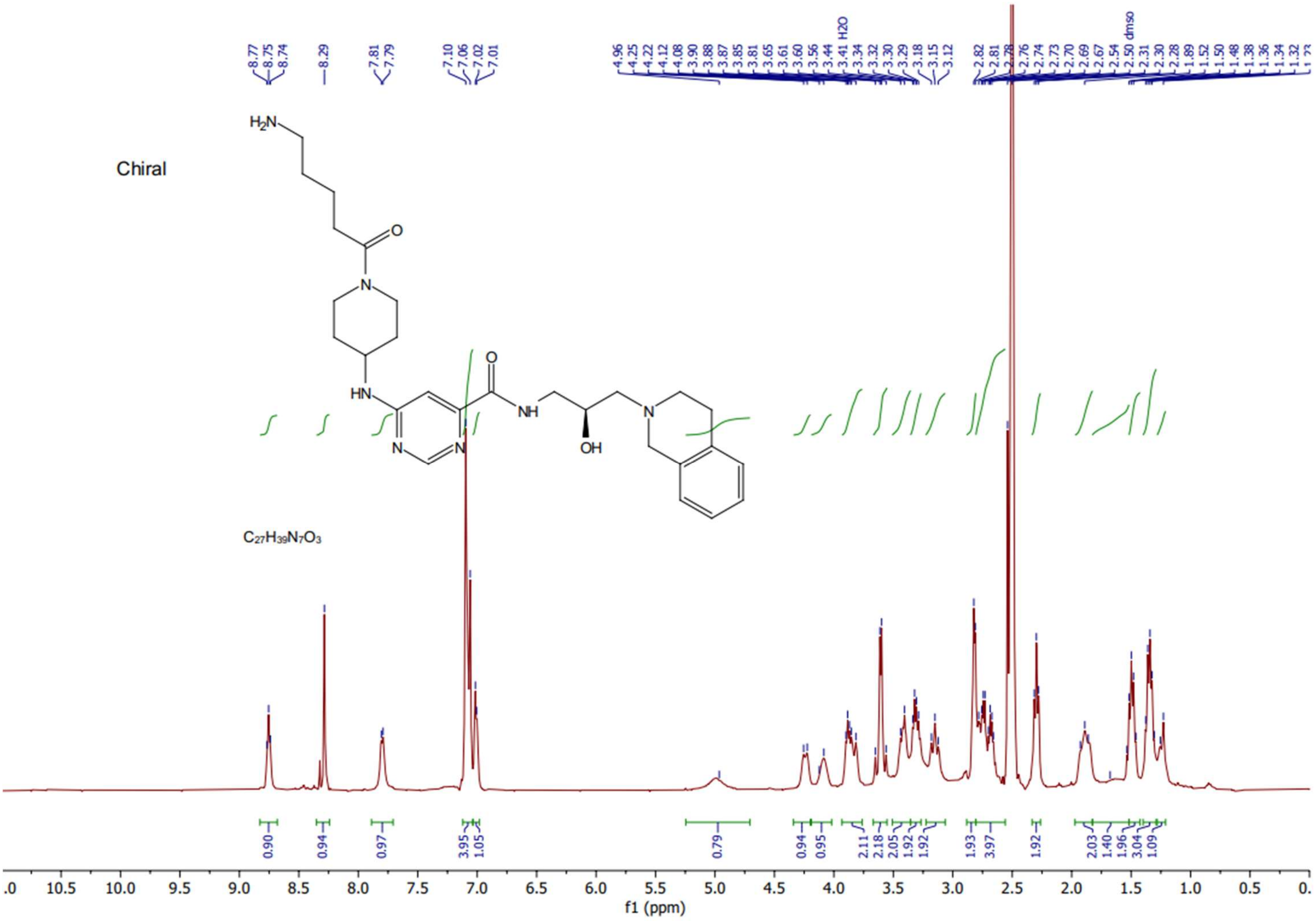

Chiral
$C_{27}H_{39}N_7O_3$
f1 (ppm)

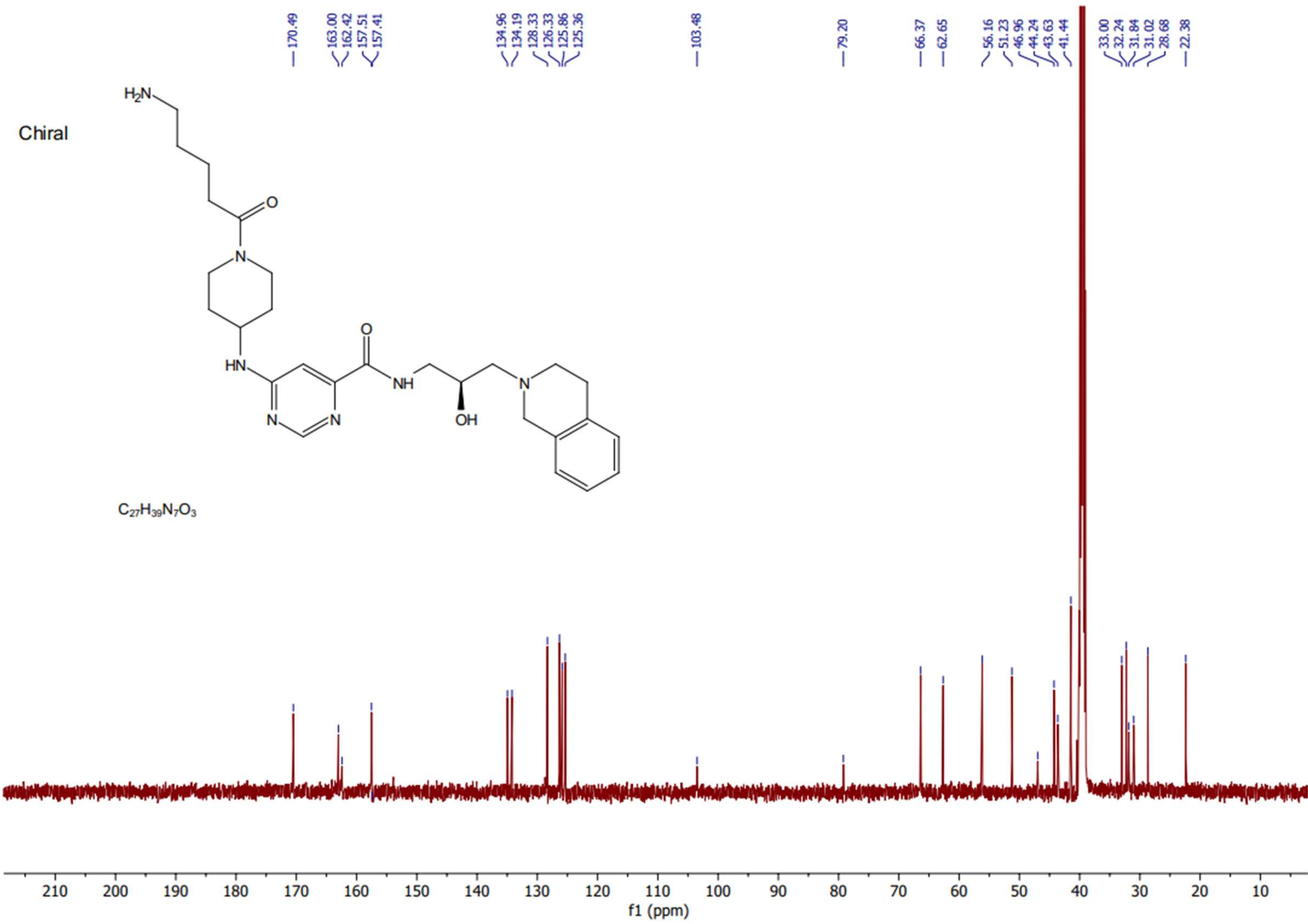
Chiral
H2N
O
N
O
HN
NH
N
N
N
OH
C27H39N7O3
170.49
163.00
162.42
157.51
157.41
134.96
134.19
128.33
126.33
125.86
125.36
103.48
79.20
66.37
62.65
56.16
51.23
46.96
44.24
43.63
41.44
33.00
32.24
31.84
31.02
28.68
22.38
210
200
190
180
170
160
150
140
130
120
110
100
90
80
70
60
50
40
30
20
10
f1 (ppm)

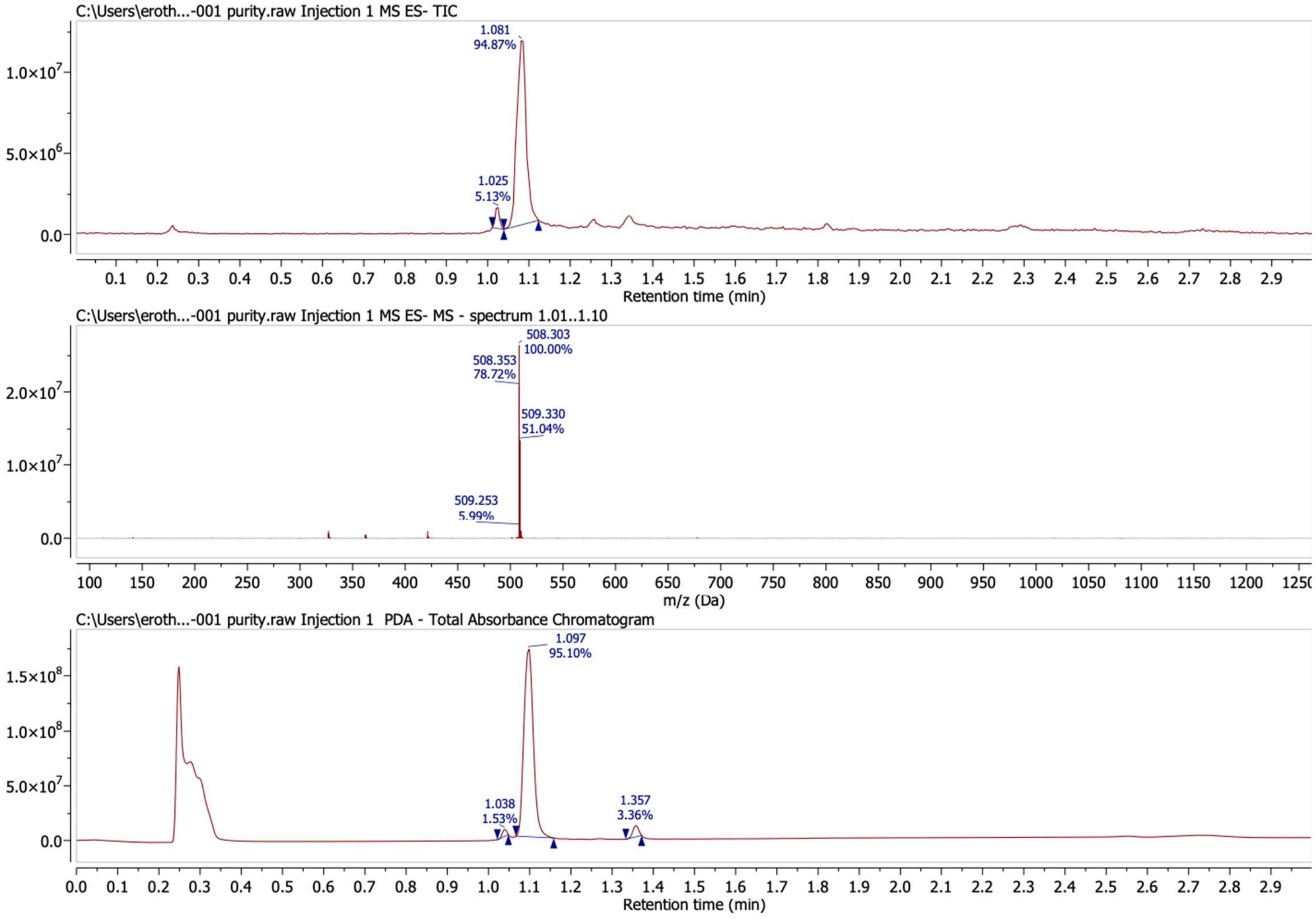
C:\Users\eroth...-001 purity.raw Injection 1 MS ES- TIC
1.081
94.87%
1.025
5.13%
Retention time (min)
C:\Users\eroth...-001 purity.raw Injection 1 MS ES- MS - spectrum 1.01..1.10
508.303
100.00%
508.353
78.72%
509.330
51.04%
509.253
5.99%
m/z (Da)
C:\Users\eroth...-001 purity.raw Injection 1 PDA - Total Absorbance Chromatogram
1.097
95.10%
1.038
1.53%
1.357
3.36%
Retention time (min)

(*S*)-6-((1-(5-(3-(5,5-difluoro-7-(1H-pyrrol-2-yl)-5H-5λ4,6λ4-dipyrrolo[1,2-c:2',1'-f][1,3,2]diazaborinin-3-yl)propanamido)pentanoyl)piperidin-4-yl)amino)-N-(3-(3,4-dihydroisoquinolin-2(1H)-yl)-2-hydroxypropyl)pyrimidine-4-carboxamide (CBH-002)

(*S*)-6-((1-(5-aminopentanoyl)piperidin-4-yl)amino)-*N*-(3-(3,4-dihydroisoquinolin-2(1*H*)-yl)-2-hydroxypropyl)pyrimidine-4-carboxamide (5.4 mg, 0.010 mmol) was dissolved in 1.0 mL of anhydrous DMF. To the stirred mixture, DIPEA (5.6 µL, 0.030 mmol) was added and stirring was continued for 10 min. To the clear colourless solution, NanoBRET® 590 SE (5 mg, 0.012 mmol) was added and the reaction was stirred to completion in the dark for 2 h. The sample was dried overnight in vacuo. The crude residue was re-solved in DMSO and subjected to reverse-phase preparative HPLC purification (Phenomenex 5 µm EVO C18 100 A, 21.2 × 150 mm column was used with a gradient program: eluent I: acetonitrile/water = 5/95 with 20 mM ammonium acetate buffer, pH 6.0; eluent II: acetonitrile/water = 80/20 with 20 mM ammonium acetate buffer, pH 6.0). Product containing fractions were concentrated in vacuo, affording the product (6.0 mg, 0.007 mmol, 69.0%) as a purple solid.

The purity was determined by LC/MS 98.2%. LC/MS (ESI-1) found 819.4 g/mol (820.45 g/mol calculated for $C_{44}H_{55}BF_2N_{10}O_3$). HR-ESI-MS $[M + H]^+$ found 821.4243 g/mol (calculated 821.4598 g/mol). ^{1}H NMR (400 MHz, DMSO) δ 11.41 (s, 1H), 8.74 (d, J = 6.0 Hz, 1H), 8.29 (d, J = 1.2 Hz, 1H), 7.92 (t, J = 5.5 Hz, 1H), 7.76 (d, J = 7.6 Hz, 1H), 7.44 (s, 1H), 7.37 (s, 1H), 7.34 (d, J = 4.5 Hz, 1H), 7.28 (s, 1H), 7.17 (d, J = 4.6 Hz, 1H), 7.12 – 7.09 (m, 3H), 7.06 (d, J = 1.2 Hz, 1H), 7.02 (s, 1H), 6.33 (d, J = 4.0 Hz, 1H), 4.97 (d, J = 4.7 Hz, 1H), 4.23 (s, 2H), 4.08 (s, 1H), 3.92 – 3.80 (m, 3H), 3.61 (d, J = 5.9 Hz, 3H), 3.18 – 3.05 (m, 4H), 2.86 – 2.64 (m, 4H), 2.38 – 2.28 (m, 1H), 1.90 (s, 1H), 1.53 – 1.38 (m, 5H), 1.36 (s, 6H), 1.24 (s, 1H).

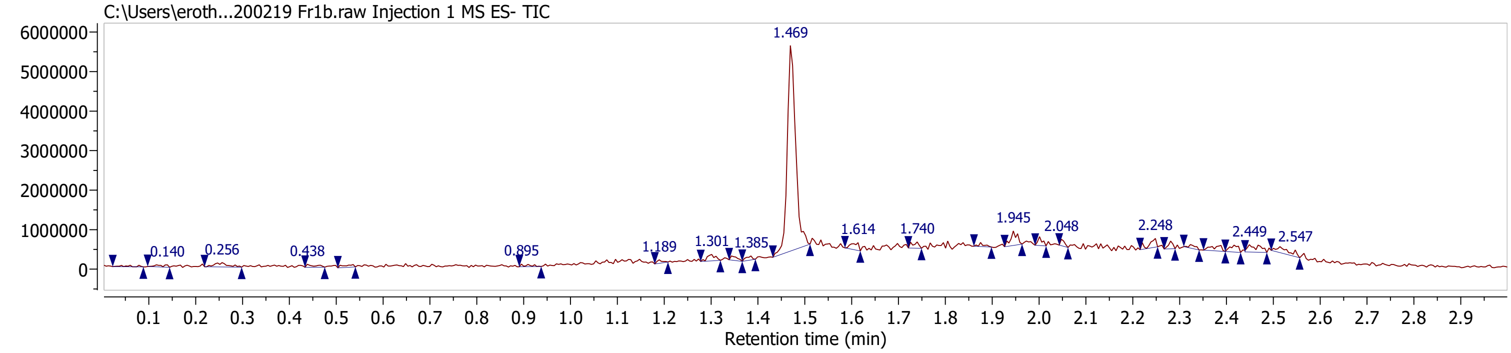
C:\Users\eroth...200219 Fr1b.raw Injection 1 MS ES- TIC
1.469
0.140
0.256
0.438
0.895
1.189
1.301
1.385
1.614
1.740
1.945
2.048
2.248
2.449
2.547
Retention time (min)

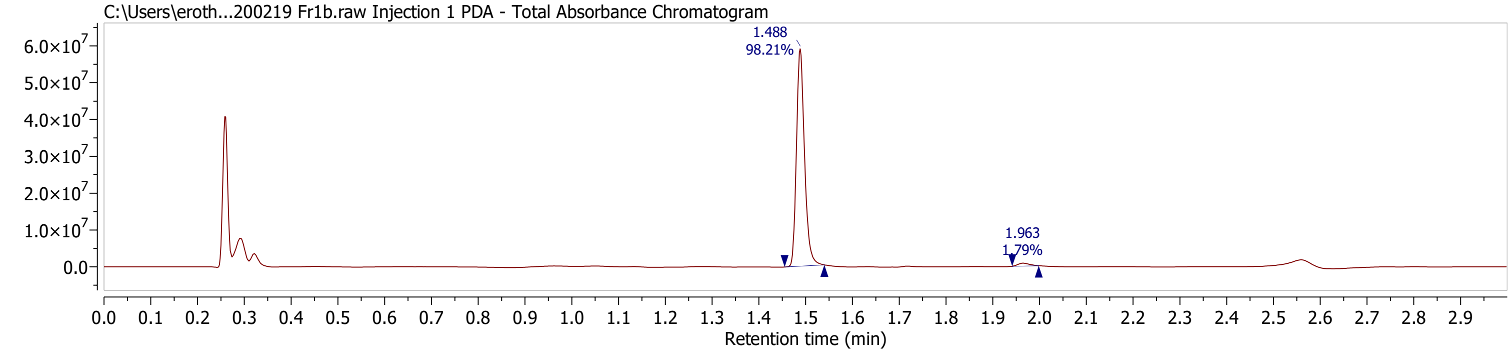
C:\Users\eroth...200219 Fr1b.raw Injection 1 PDA - Total Absorbance Chromatogram
1.488
98.21%
1.963
1.79%
Retention time (min)

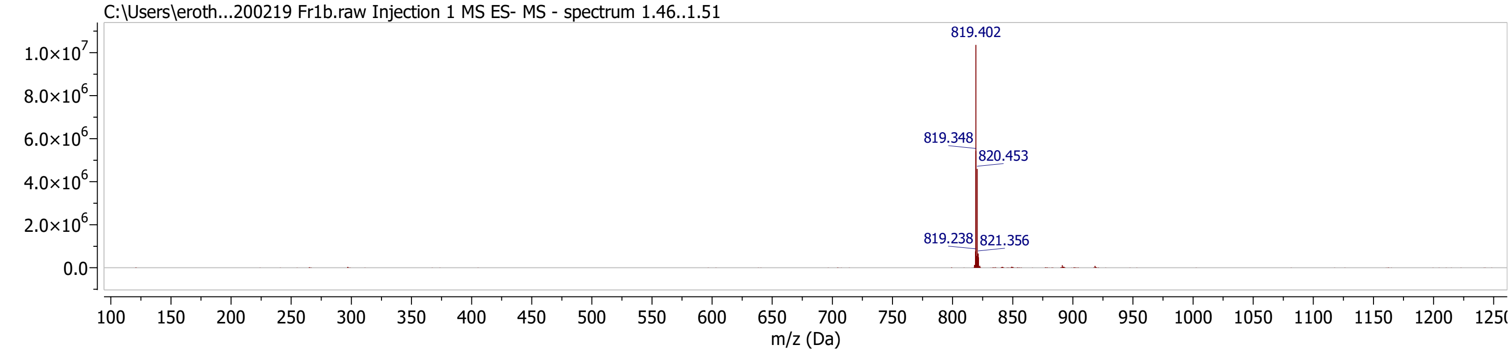
C:\Users\eroth...200219 Fr1b.raw Injection 1 MS ES- MS - spectrum 1.46..1.51
819.402
819.348
820.453
819.238
821.356
m/z (Da)

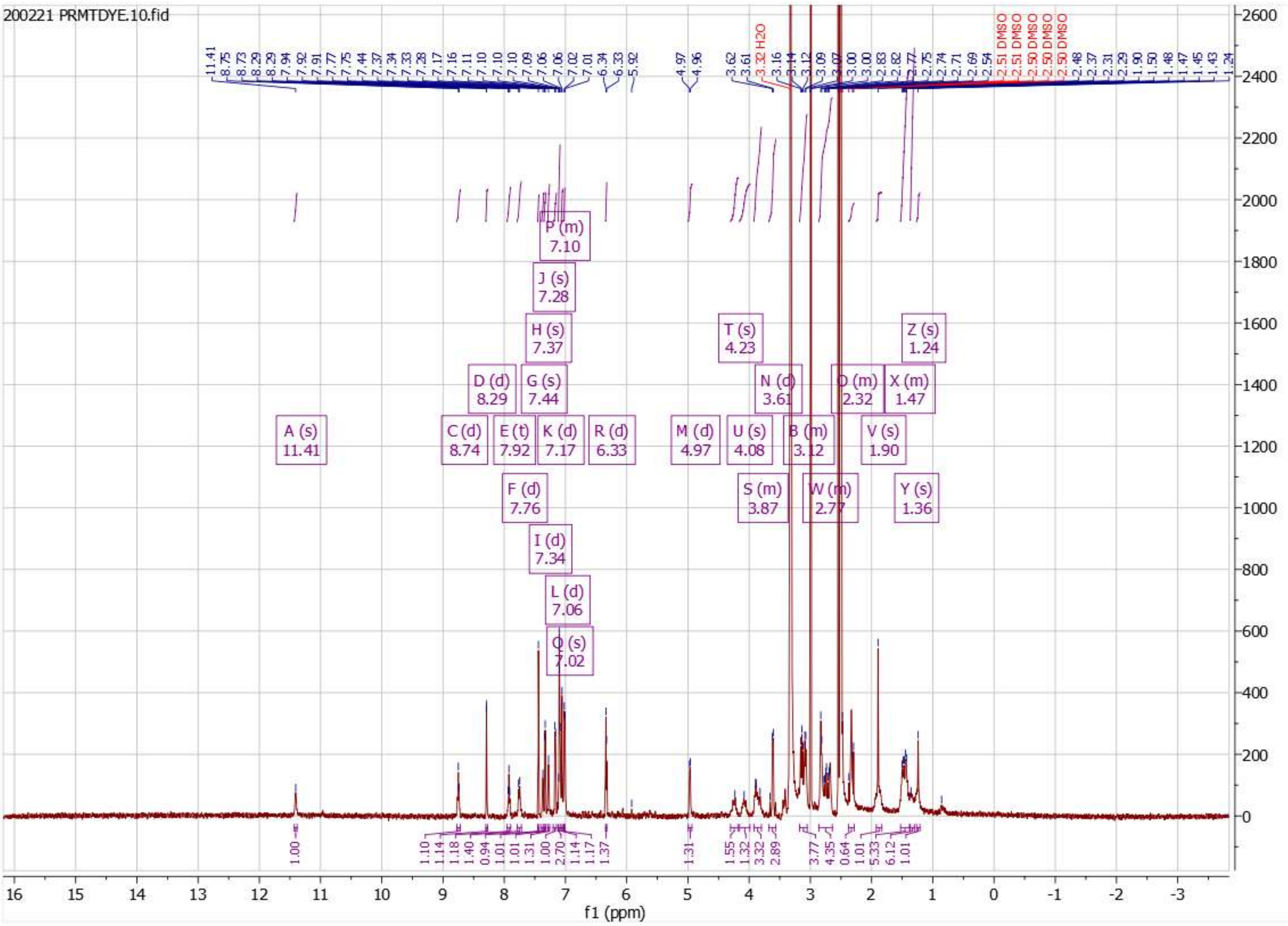
200221 PRMTDYE.10.fid
A (s) 11.41
C (d) 8.74
D (d) 8.29
E (t) 7.92
F (d) 7.76
G (s) 7.44
H (s) 7.37
I (d) 7.34
J (s) 7.28
K (d) 7.17
L (d) 7.06
P (m) 7.10
Q (s) 7.02
R (d) 6.33
M (d) 4.97
T (s) 4.23
U (s) 4.08
S (m) 3.87
N (d) 3.61
B (m) 3.12
W (m) 2.77
O (m) 2.32
V (s) 1.90
X (m) 1.47
Y (s) 1.36
Z (s) 1.24
3.32 H2O
2.51 DMSO
2.50 DMSO
f1 (ppm)

2. Supplementary Figures and Tables

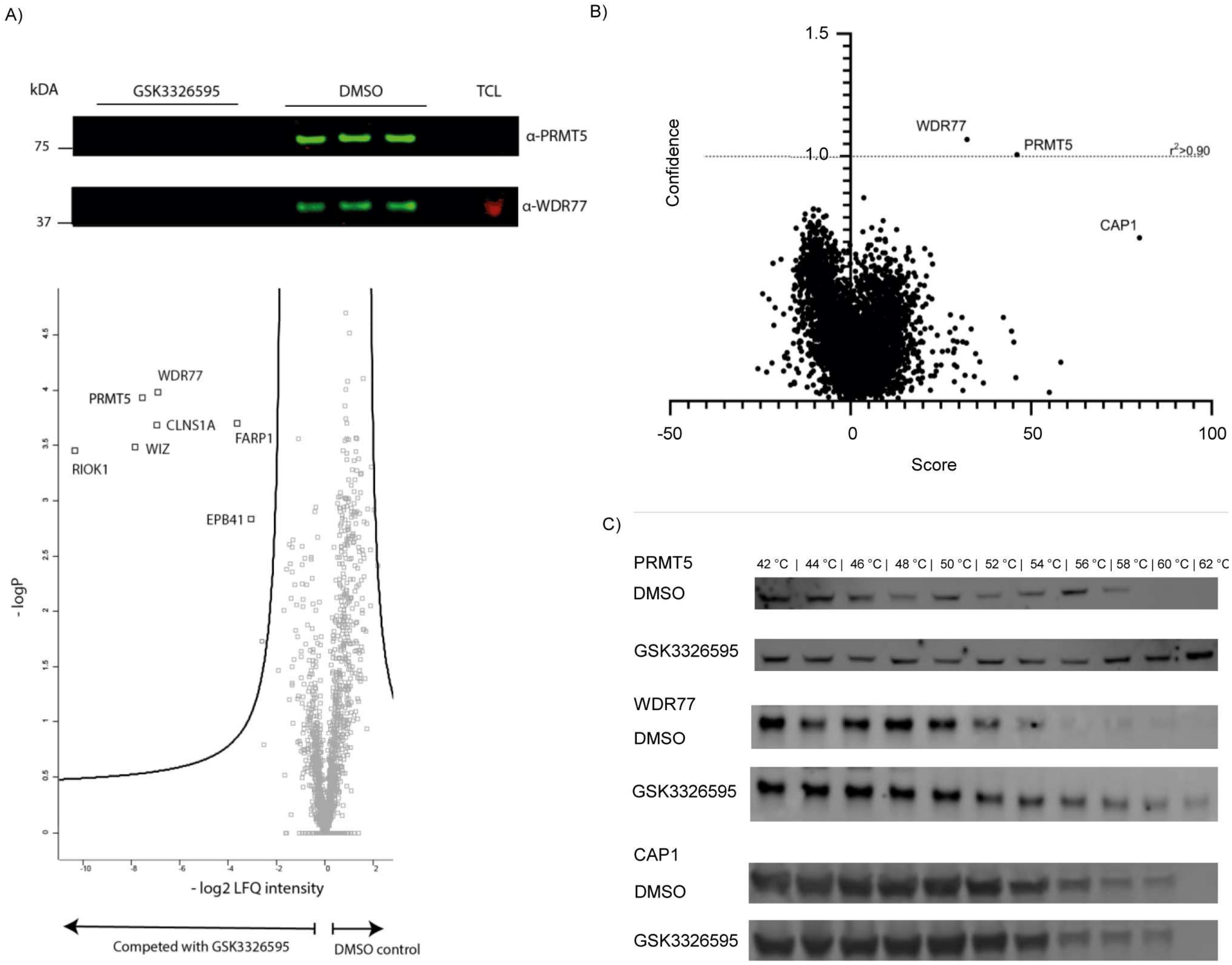

Supplementary Figure S1. Proteome-wide specificity of GSK3326595 as determined by chemical proteomics. A) Western blot analysis of competitive PRMT5 engagement by affinity probe CBH-001. Competition with either parent inhibitor or DMSO in KMS11 lysate (n=3) shows inhibitor-dependent enrichment of PRMT5 (72 kDa, green) and WDR77 (36 kDa, green) (TCL, total cell lysate). Red band indicates GAPDH which was used as TCL loading control (n=1). Volcano plot of CBH-001-enriched proteome from KMS11 cell lysate (n=3), with targets significantly competed by 20 µM GSK3326595 versus DMSO control (FDR = 0.05, S0 = 0.2). B) 2D thermal profiling results for GSK3326595 identify the methylosome complex comprising PRMT5 and WDR77. For each protein, the score indicates the volume under the surface of stabilization as a 2-dimensional function of temperature and compound concentration as calculated by the algorithm, and confidence is $-\log(1-r^2)$ of the fitted surface. MATLAB's poly31 model was used to fit a surface equation to the stabilisation data with a least-squares model, resulting in a fitted surface with an R-squared value that gave a measure of the goodness-of-fit of the model, as well as the relative integral volume under the fitted surface, giving a measure of the magnitude of stabilisation that is, in principle, corrected for outliers. By -log transforming (1-R2) and plotting the data, a plot akin to a volcano plot was obtained, depicting both the magnitude and a measure of goodness of model fit. By setting the

cut-off at R2 of 0.9, two proteins appear as hits from the assay, methylosome protein 50 (WDR77/MEP50) and, as mentioned above, PRMT5, with another protein, adenylyl cyclase associated protein 1 (CAP1) being a possible hit, although its goodness-of-fit is far below the cut-off, suggesting the high magnitude could be a false positive.C) Validation of PRMT5 and WDR77 via Western blot CETSA using 5 μM GSK3326595 (n=1). Uncropped blots are provided as a Source Data file.

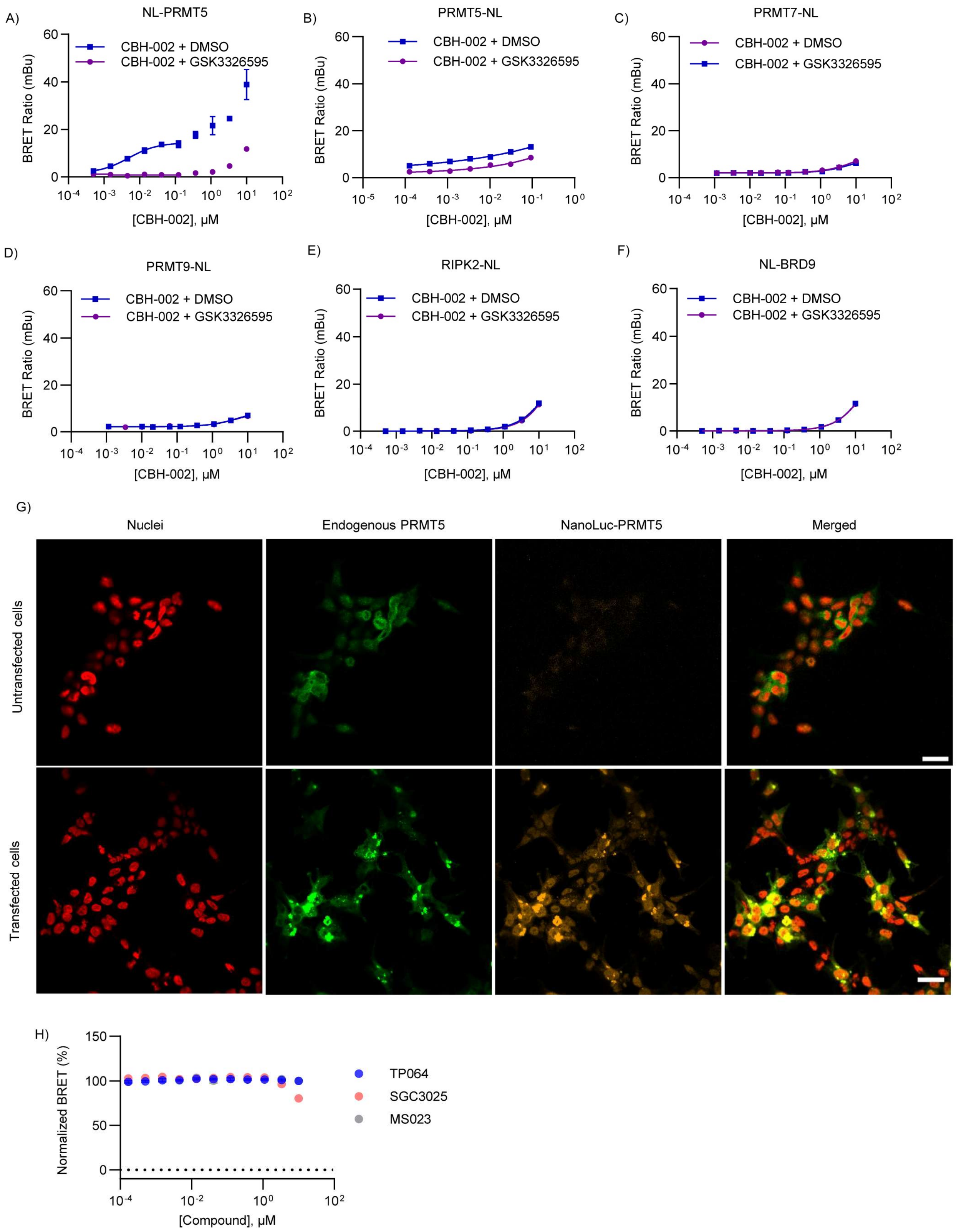

Supplementary Figure S2. Validation of BRET probe CBH-002. A-F) Affinity curves for BRET probe CBH-002 in HEK293 cells transfected with various NanoLuc constructs. CBH-002 was titrated against 10 µM GSK3326595 or DMSO. Data are the mean ± SEM of indicated number of independent experiments for each condition. A) NL-PRMT5 fusion vector with

empty carrier DNA (no WDR77 co-expression) (n=1). B) PRMT5-NL fusion vector with empty carrier DNA (no WDR77 co-expression) (n=1). C) PRMT7-NL fusion vector (n=3) D) PRMT9-NL fusion vector (n=3). E) RIPK2-NL fusion vector (n=3), cytosolic control. F) NL-BRD9 fusion vector (n=3), nuclear control. G) Subcellular localization and co-localization of endogenous PRMT5 and NL-PRMT5. Stains shown in untransfected and transfected HEK293 cells in separate and merged channels. Scale bars are 20 µm. H) Representative EC_{50} curves for negative control compounds TP064 (PRMT4), SGC3025 (PRMT7) and MS023 (PRMT1/3/4/6/8) in HEK293 cells expressing NL-PRMT5 fusion DNA and unlabelled WDR77 DNA. Data are the mean ± SEM of 3 independent experiments (n=3). Source data are provided as a Source Data file.

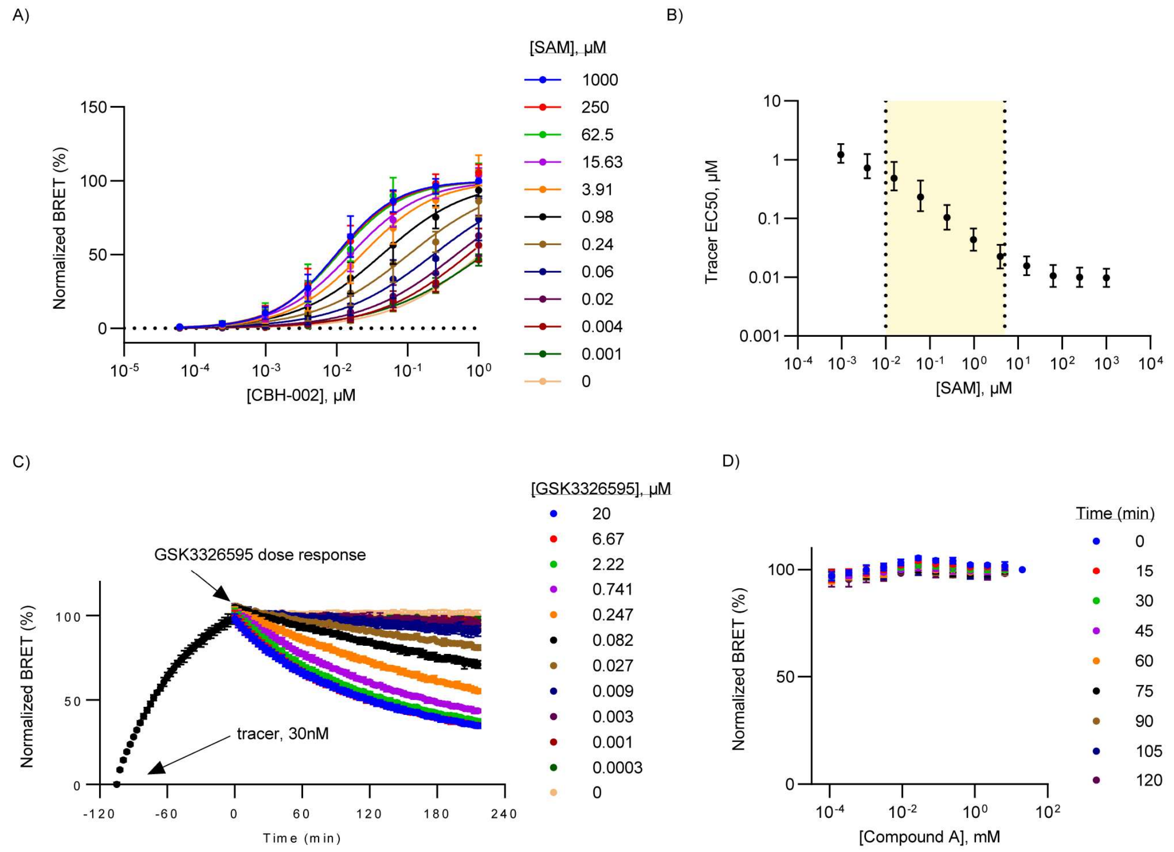

Supplementary Figure S3. BRET probe sensitivity to intracellular SAM and MTA, related to Figure 3. A) Titration matrix of BRET probe CBH-002 with SAM in HEK293 cell lysate. Cells expressing NLuc-PRMT5 were lysed and treated with an 11 pt 4-fold series of SAM and an 8 pt 2-fold series of CBH-002. Lysate was incubated at room-temperature for 30 min prior to reading BRET. Data are the mean ± SEM from 3 experiments (n=3). B) Plot of CBH-002 EC_{50} (from Fig. S3A) versus SAM concentration. Yellow shaded portion indicates linear range of SAM concentration as detected by BRET probe. Data are the mean ± 95% CI. C) Time course data of BRET probe binding kinetics in live HEK293 cells. Cells expressing NLuc-PRMT5 were treated with 30 nM CBH-002 and data was acquired in 3 min intervals for 2 h at room temperature. Then, a 20 µM, 3-fold, 11 pt titration series of GSK3326595 was added to the cells and tracer dissociation was measured at 3-minute intervals over the course of 3.5 h. Data are the mean ± SD of 4 technical replicates (n=4). D) Representative data showing BRET probe signal as a function of MAT2A inhibition and time at room temperature. HEK293 cells expressing NLuc-PRMT5 were pre-treated with CBH-002 for 2 h prior to treatment with a titration series of MAT2A inhibitor (Compound A). The plate was read in 1 h intervals at room temperature. Data are the mean ± SD of 3 technical replicates (n=3). Source data are provided as a Source Data file.

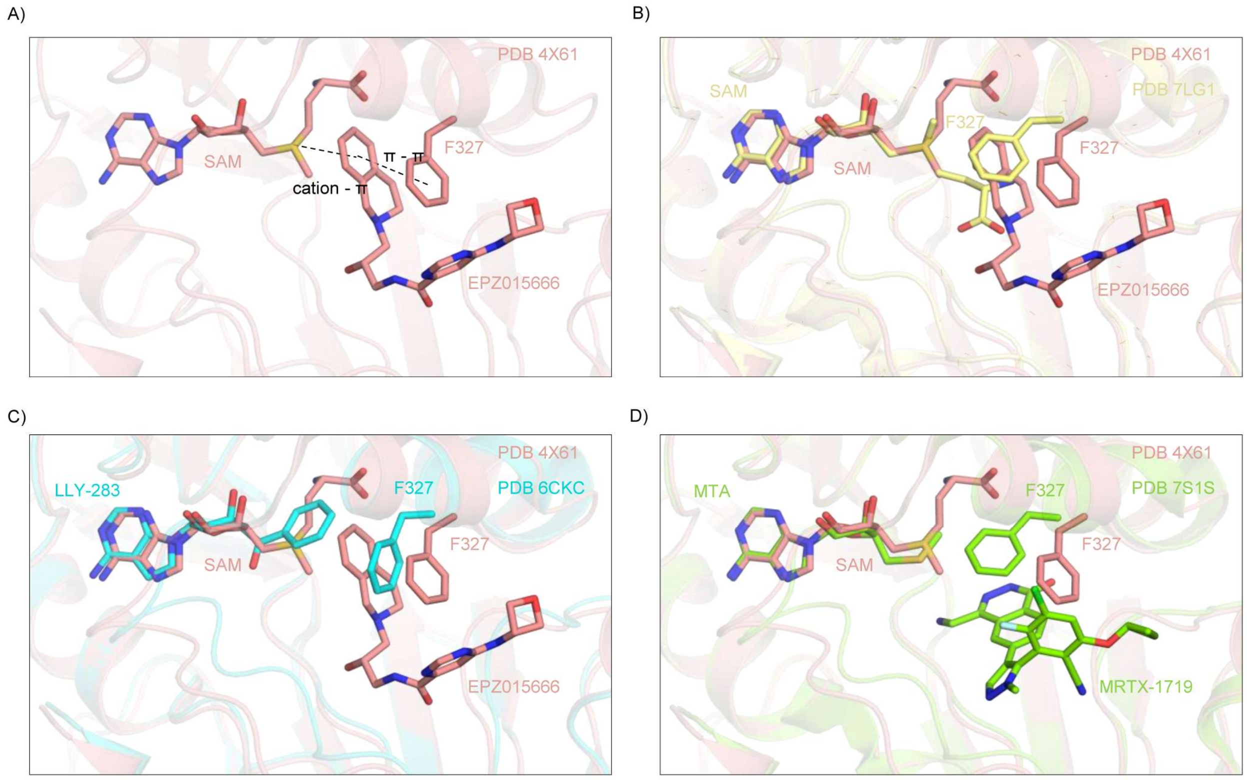

Supplementary Figure S4. Co-crystal structures reveal the distinct binding modes of SAM, EPZ015666 (GSK3235025), LLY-283, MTA and MRTX1719 to PRMT5. A) Stacking interactions are mediated between SAM and tetrahydroquinoline scaffold of EPZ015666, as well as between EPZ015666 and F327 (PDB: 4X61, marine). B) The binding of EPZ015666 induces a conformational change in F327 compared to the structure of PRMT5 bound by SAM alone (PDB: 7L1G, yellow). C) Compared to the SAM-mimetic inhibitor LLY-283 (PDB: 6CKC, blue), binding of EPZ015666 (PDB: 4X61, marine) necessitates repositioning of F327. D) Unlike EPZ015666, PRMT5 bound by MRTX1719 and MTA (PDB: 7S1S, green) exhibits a preferential positioning of F327 similar to that observed in structures where PRMT5 is bound by SAM alone or LLY-283.

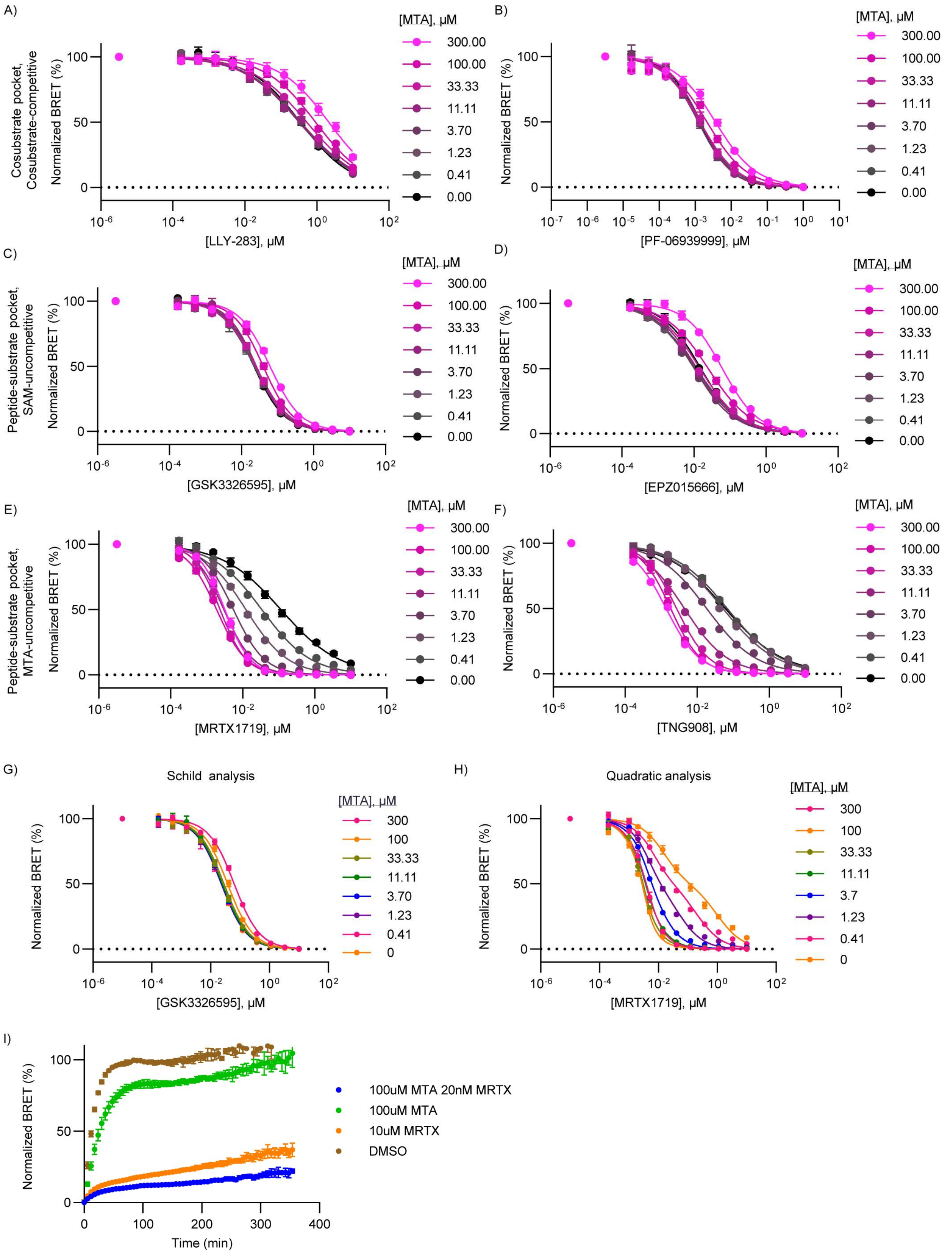

Supplementary Figure S5. Influence of MTA on PRMT5 inhibitor engagement, related to Figure 4. A-F) Full titration matrices for PRMT5 inhibitors and MTA shown in figure 4. HEK293 cells were treated with 30 nM BRET probe, a 100 µM, 7 pt, 3-fold series of MTA, and an 11 pt, 3-fold series of PRMT5 inhibitor for 2 h prior to BRET measurements. Data are

the mean ± SEM of 3 independent experiments (n=3). G) Representative global fit using the Gaddum/Schild EC_{50} shift equation. The global fitting is shown for GSK3326595 and was used for all compounds where the Hill slope did not vary significantly as a function of MTA (PF06939999, EPZ015666, LLY283). H) Representative quadratic fit for MRTX1719 as a function of MTA. Compounds where addition of MTA significantly changed the Hill slope (TNG908 and MRTX1719) were analyzed using this method. I) Kinetic washout data illustrating the influence of MTA on MRTX1719 residence time in HEK293 cells. HEK293 cells were treated with either 0 µM or 100 µM MTA, as well as a saturating dose of MRTX1719 under those MTA conditions for 2 h at 37 °C to induce complex formation. Cells were then washed to remove excess compound, while MTA concentrations were kept constant, before being treated with 300 nM CBH-002. Plates were immediately read in a luminometer set at 37 °C for 6 h. Data are the mean ± SEM of 3 independent experiments (n=3). Source data are provided as a Source Data file.

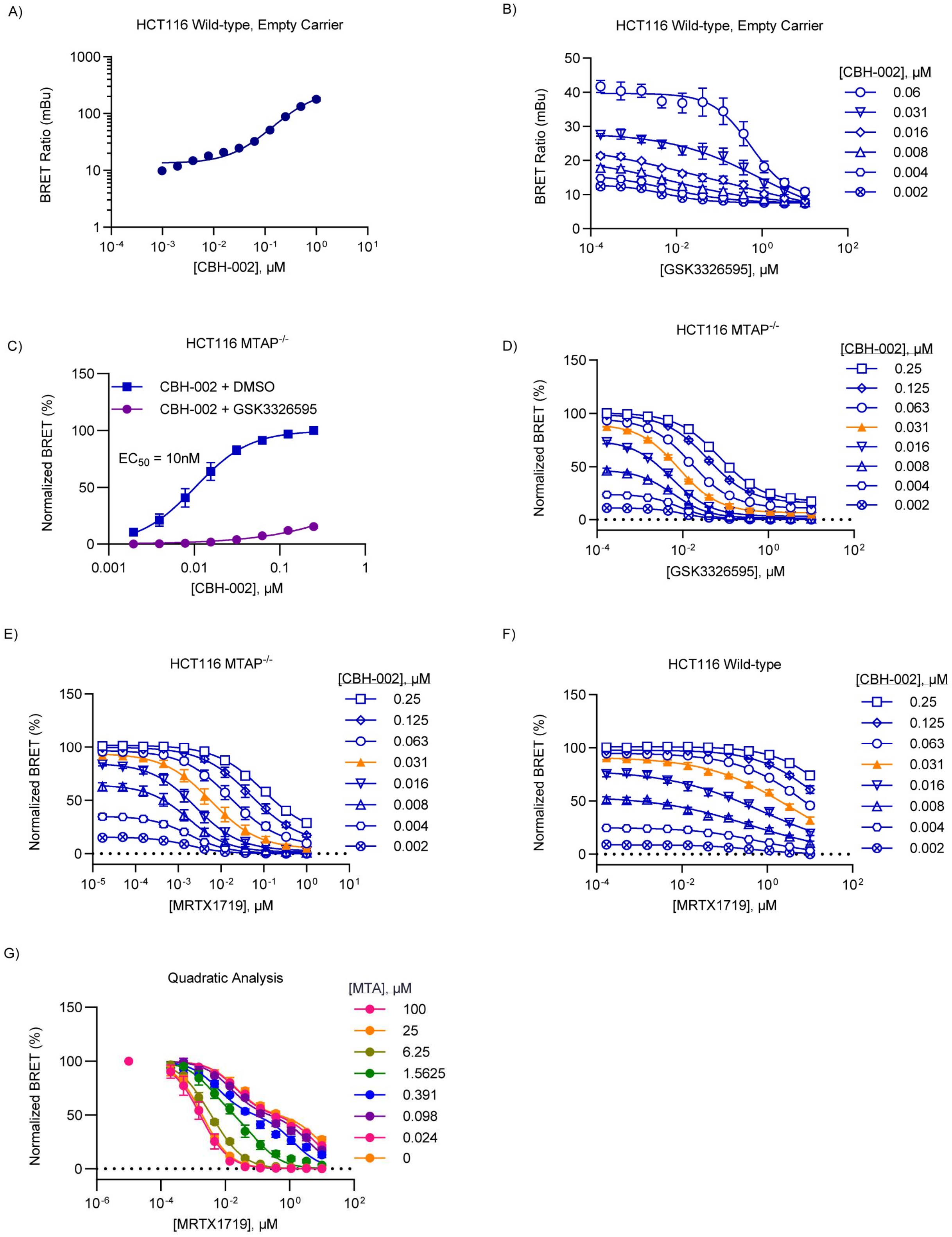

Supplementary Figure S6. Surveying synthetic lethal engagement of PRMT5-MTA complexes in MTAP-knockout cells, related to Figure 5. A) Affinity curve for BRET probe CBH-002 in HCT116 MTAP$^{-/-}$ cells transfected with NLuc-PRMT5 and unlabelled WDR77 DNA. Data are plotted on log-scale to show biphasic behaviour and fit with a 4-parameter regression curve (see methods). Data are the mean ± SD of 1 independent experiment with 4-

technical replicates (n=1). B) Matrix of CBH-002 and GSK3326595 in HCT116 MTAP$^{-/-}$ cells expressing NLuc-PRMT5 DNA and empty carrier plasmid. Data are the mean ± SEM of 3 independent experiments (n=3) and are fit with a 4-parameter regression curve (see methods). C-D) BRET probe CBH-002 validation data in HCT116$^{-/-}$ cells expressing NLuc-PRMT5 and unlabelled WDR77 DNA. HCT116 MTAP$^{-/-}$ cells were treated with an 11 pt 3-fold series of GSK3326595 and an 8 pt, 2-fold titration series of CBH-002 for 2 h before BRET was measured. Data are the mean ± SEM of 3 independent experiments (n=3). Data was normalized and fit with a 2-parameter regression analysis (C) or 4-parameter regression analysis (D, see methods). Data is plotted as both an affinity curve (C) and compound matrix (D). Optimal BRET probe concentration shown in orange. E-F) Full titration matrices of CBH-002 and MRTX1719 in MTAP$^{-/-}$ (E) and HCT116 MTAP wild-type (F) cells. HCT116 cells co-expressing NLuc-PRMT5 and untagged WDR77 DNA were treated with 11 pt, 3-fold titrations of compound and 8 pt, 2-fold titrations of BRET probe CBH-002 for 2 h prior to BRET measurement. Curves were normalized and fit with a 4-parameter regression model (see methods). Data are the mean ± SEM of 3 independent experiments (n=3). Optimal BRET probe concentration shown in orange. G) Quadratic fit for MRTX1719 as a function of MTA in HCT116 MTAP wild-type cells. Source data are provided as a Source Data file.

Assay type	GSK3326595[1]	EPZ015666[2,3]	LLY-283[4]	PF-06939999[5,6]	MRTX1719[7,8]	TNG908[9]
Biochemical (nM)	6.2 [a]	4 – 11[d]	22 [d]	n.a.	3.6 – 20.4 [m]	21.2 – 262 [p]
SPR (pM)	n.a.	n.a.	6000 [g]	5.8 [j]	0.140 – 9.4 [n]	n.a.
In-Cell Western (nM)	0.3 – 14[b]	3 – 56 [e]	25 [h]	1.1 [k]	8 – 653 [o]	9 – 135 [r]
Proliferation (nM)	5.7 – 106 [c]	62 [f]	3 – 2500 [i]	3.87 – 5.14 [l]	12 – 890 [q]	100 – 1500 [s]
NanoBRET EC_{50} (95% CI, nM)	19.9 (18 – 21)	26.9 (24 – 29)	467.5 (409 – 533)	1.7 (1.5 – 1.9)	80 (68 – 93)	135 (117 – 155)

Supplementary Table S1. Comparison of literature data and NanoBRET TE assay results (n=3). a) HTRF assay monitoring monomethylation of H4R3 on H4 peptide by PRMT5:MEP50 (IC_{50}); b) In-Cell Western (ICW) sDMA Western, various cell lines (EC_{50}); c) Long Term Proliferation (LTP) assay, various cell lines (IC_{50}); d) Radioactive assay monitoring methyl transfer from 3H-SAM to peptide substrate (IC_{50}); e) Symmetric arginine methylation of SmD3 in Z-138 cells (IC_{50}); f) Proliferation assay in Z-138 cells (IC_{50}); g) Surface Plasmon Resonance (SPR) with PRMT5:MEP50 complex (K_d); h) Symmetric dimethylation of SmBB' in MCF7 cells (EC_{50}); i) Cancer cell line proliferation assay (IC_{50}); j) Single-cycle kinetic analysis SPR (K_d); k) Proliferation assay in A427, measurement of SDMA levels (IC_{50}); l) Antiproliferation IC_{50} value for NCI-H1975; m) Radioactivity assay PRMT5–MEP50 transfer of the methyl group from S-adenosyl-l-[methyl-3H]methionine generates a radiolabelled histone H4 (MTA – SAM IC_{50}); n) PRMT5/MEP50 4-point SPR assay run in chaser mode to determine k_{off} with 20 µM MTA or 20 µM SAM preincubation (MTA – SAM k_{off}); o) MTAP-del and MTAP WT SDMA assay (MTAP del – MTAP WT cells IC_{50}); p) Radioactive flashplate assay (IC_{50}); q) 10 day cell viability assay in MTAP del and MTAP WT cell lines (MTA – APO IC_{50}); r) SDMA in-cell western (MTAP del – MTAP WT cells EC_{50}); s Cell viability assay using CellTiter-Glo assay in HAP1 MTAP-isogenic cells (MTAP deleted – MTAP Wild-type cells IC_{50}); n. a., not available.

	LLY283			PF06939999			GSK3326595			EPZ015666			MRTX1719			TNG908		
[MTA]	EC_{50}, nM	EC_{50} 95% CI, nM	Hill slope	EC_{50}, nM	EC_{50} 95% CI, nM	Hill slope	EC_{50}, nM	EC_{50} 95% CI, nM	Hill slope	EC_{50}, nM	EC_{50} 95% CI, nM	Hill slope	EC_{50}, nM	EC_{50} 95% CI, nM	Hill slope	EC_{50}, nM	EC_{50} 95% CI, nM	Hill slope
300 μM	2147	1654 - 2836	0.64	3.6	2.9 - 4.5	0.76	62.0	57 - 68	1.00	61.6	56 - 67	0.87	3.2	2.9 - 3.4	1.22	1.4	1.3 - 1.4	0.93
100 μM	919.6	789 - 1074	0.62	2.1	1.8 - 2.5	0.79	40.2	37 - 43	1.05	24.9	23 - 27	0.72	1.9	1.8 - 2	1.05	1.8	1.7 - 2	1.03
33.33 μM	523.2	468 - 585	0.56	1.4	1.2 - 1.5	0.84	25.0	23 - 27	0.93	14.7	13 - 16	0.76	2.3	2.1 - 2.5	1.19	2.7	2.5 - 2.9	0.96
11.11 μM	356.3	302 - 420	0.55	1.4	1.3 - 1.5	0.99	25.9	23 - 29	1.07	12.1	11 - 13	0.77	3.0	2.9 - 3.1	1.06	4.4	4.2 - 4.7	0.72
3.7 μM	322.2	278 - 374	0.56	1.2	1.1 - 1.4	0.93	24.7	23 - 26	1.02	10.2	9.5 - 11	0.77	5.5	5.2 - 5.7	1.00	18.3	16 - 20	0.63
1.23 μM	340.7	293 - 396	0.57	1.4	1.3 - 1.5	0.98	23.3	21 - 26	0.98	10.6	9.8 - 11	0.71	12.2	11 - 13	0.74	52.1	47 - 57	0.58
0.41 μM	341.5	299 - 390	0.55	1.3	1.2 - 1.5	0.89	22.8	20 - 26	0.96	11.8	11 - 12	0.70	33.7	30 - 38	0.64	68.4	63 - 74	0.58
0 μM	347.6	290 - 418	0.62	1.3	1.2 - 1.4	0.97	22.3	21 - 24	1.02	15.6	14 - 18	0.81	114.3	96 - 136	0.53	60.6	55 - 67	0.54

Supplementary Table S2. PRMT5 inhibitor potency as a function of MTA concentration, related to Figure 4 and Figure S5. Data are the mean of 3 independent experiments (n=3).

Compound	Analysis type[1]	Hill slope[2]	Schild slope[3]	A2, μM[4]	EC_{50}, nM (MTA = 0)[5]	K_b (MTA), μM[6]
LLY-283	Global/Schild	0.55 (0.51 - 0.59)	1.1 (0.93 - 1.31)	61 (46-79)	365 (290 - 470)	54 (45 - 55)
PF-06939999	Global/Schild	1	1.15 (0.88 - 1.51)	172 (137 – 211)	1.3 (1.2 - 1.4)	162 (133 - 202)
GSK3326595	Global/Schild	1	0.92 (0.75 - 1.12)	158 (126 – 199)	23 (22 - 24)	170 (148 - 196)
EPZ015666	Global/Schild	0.75 (0.73 - 0.78)	1.3 (1.15 - 1.47)	97 (84-112)	11 (10.4 - 11.7)	75 (67 - 85)
			[MTA] = 0[9]		[MTA] = 100**, 300 uM[10]	
Compound	Analysis type[7]	Enzyme total, nM[8]	K_i, nM	K_{ii}, nM	K_i, nM	K_{ii}, nM
MRTX1719	Quadratic/Morrison	1.6 (1.1 - 2.1)	1000 (900 - 1300)	12.7 (1.1 - 1.5)	2.6 (0.1 - 3.7)**	0.4 (0.1 - 3.7)**
TNG908	Quadratic/Morrison	1.6	500 (400 - 700)	7 (5 - 10)	2.3 (1.2 - 3.5)	0.13 (0.39 - 0.44)

Supplementary Table S3. Modeling parameters for PRMT5i binding analysis, related to Figure 4 and Figure S5.

[1]Data were globally fit to the Gaddum/Schild EC_{50} shift equation using GraphPad Prism 10 $EC50 = 10^{LogEC50}$.

$$Antag = 1 + \frac{B}{(10^{-pA2})^S}$$

$$LogEC = Log(EC50.Antag)$$

$$Y = Y_{min} + \frac{Y_{max} - Y_{min}}{1 + 10^{(LogEC-X)h}}$$

Where EC_{50} is the compound affinity at [MTA]=0, h is the Hill Slope, pA2 is the negative logarithm of the concentration of MTA needed to shift the curve by a factor of 2.

[2]The EC_{50} shift equation uses a constant Hill slope across the concentration response curves at each [MTA]. For PF06939999 and GSK3326595 the Hill slope was constrained to 1, based on the analysis of each individual data set (Table S2). For LLY283 and EPZ015666 the Hill slope was not constrained and global fitting returned values similar to those observed from individual data set analysis.

[3]The Schild slope (S) quantifies how well the effect of MTA on compound binding approximates competitive binding. For all compounds the slope was 1 within the CL limits except for EPZ015666 where a value slightly above 1 was observed.

[4]A2 is the concentration of MTA needed to shift the dose response curve by a factor of 2.

[5]EC_{50} is the value obtained at [MTA] = 0.

[6]Constraining the Schild slope to 1 yields K_b, the apparent affinity of MTA for PRTM5.

[7]Date were globally fit to an equation containing two quadratic functions assuming two sites with differentially affinity for the inhibitor and that at high [MTA] the apparent affinity of the compound for PRMT5 is under tight binding conditions which requires analysis using the Morrison equation that explicitly uses $[E]_t$ (total enzyme concentration) and $[I]_t$ (total inhibitor concentration).11

$$Y = 50\left(1 - \frac{([E]_T + X + K_i) - \sqrt{([E]_T + X + K_i)^2 - 4[E]_T X}}{2[E]_T}\right) + 50\left(1 - \frac{([E]_T + X + K_{ii}) - \sqrt{([E]_T + X + K_{ii})^2 - 4[E]_T X}}{2[E]_T}\right)$$

[8]Fitting of the MRTX1719 data yielded a total enzyme concentration of 1.6 nM which was uses as input for fitting the TNG908 data.

[9]Data fitting yields two binding constants K_i and K_{ii}.

[10]For MRTX1719 fitting of the data for [MTA] = 300 μM was unstable and the reported values are for concentrations of [MTA] up to 100 μM.

	MTAP WT		MTAP-/-	
	GSK3326595	MRTX1719	GSK3326595	MRTX1719
HillSlope (95% CI)	0.9 (0.8-1.0)	0.4 (0.4-0.5)	0.9 (0.8-1.0)	0.8 (0.7-1.0)
EC_{50}, nM (95% CI)	9.3 (8-11)	> 1000	8.2 (7-9)	5.9 (4-8)

Supplementary Table S4. Fit curve parameters for HCT116 cells, related to Fig. 5C.

[MTA]	EC_{50}, nM (95% CI)	HillSlope (95% CI)
100	1.6 (1.3 - 2.0)	1.1 (0.9 - 1.3)
25	1.9 (1.7 - 2.1)	1.1 (1.0-1.1)
6.25	3.6 (3.2 - 3.9)	0.89 (0.82 - 0.96)
1.5	19 (15 - 24)	0.65 (0.57 - 0.75)
0.4	77 (61 - 98)	0.43 (0.39 - 0.48)
0.1	23 (18 - 29)	0.43 (0.39 - 0.47)
0.02	371 (312 - 441)	0.41 (0.38 - 0.44)
0	498 (367 - 686)	0.37 (0.32 - 0.42)

Supplementary Table S5. Fit curve parameters for MTA titration in HCT116 WT cells, related to Fig. 5D.

Supplementary References

1:Gerhart, S. V.; Kellner, W. A.; Thompson, C.; Pappalardi, M. B.; Zhang, X.-P.; Montes de Oca, R.; Penebre, E.; Duncan, K.; Boriack-Sjodin, A.; Le, B., Activation of the p53-MDM4 regulatory axis defines the anti-tumour response to PRMT5 inhibition through its role in regulating cellular splicing. Scientific reports 2018, 8 (1), 9711.

2:Duncan, K. W.; Rioux, N.; Boriack-Sjodin, P. A.; Munchhof, M. J.; Reiter, L. A.; Majer, C. R.; Jin, L.; Johnston, L. D.; Chan-Penebre, E.; Kuplast, K. G., Structure and property guided design in the identification of PRMT5 tool compound EPZ015666. ACS medicinal chemistry letters 2016, 7 (2), 162-166.

3:Scheer, S.; Ackloo, S.; Medina, T. S.; Schapira, M.; Li, F.; Ward, J. A.; Lewis, A. M.; Northrop, J. P.; Richardson, P. L.; Kaniskan, H. Ü., A chemical biology toolbox to study protein methyltransferases and epigenetic signaling. Nature communications 2019, 10 (1), 19.

4:Bonday, Z. Q.; Cortez, G. S.; Grogan, M. J.; Antonysamy, S.; Weichert, K.; Bocchinfuso, W. P.; Li, F.; Kennedy, S.; Li, B.; Mader, M. M., LLY-283, a potent and selective inhibitor of arginine methyltransferase 5, PRMT5, with antitumor activity. ACS medicinal chemistry letters 2018, 9 (7), 612-617.

5:Jensen-Pergakes, K.; Tatlock, J.; Maegley, K. A.; McAlpine, I. J.; McTigue, M.; Xie, T.; Dillon, C. P.; Wang, Y.; Yamazaki, S.; Spiegel, N., SAM-competitive PRMT5 inhibitor PF-06939999 demonstrates antitumor activity in splicing dysregulated NSCLC with decreased liability of drug resistance. Molecular cancer therapeutics 2022, 21 (1), 3-15.

6:Kumar, D.; Jain, S.; Coulter, D. W.; Joshi, S. S.; Chaturvedi, N. K., PRMT5 as a Potential Therapeutic Target in MYC-Amplified Medulloblastoma. Cancers (Basel) 2023, 15 (24).

7:Smith, C. R.; Aranda, R.; Bobinski, T. P.; Briere, D. M.; Burns, A. C.; Christensen, J. G.; Clarine, J.; Engstrom, L. D.; Gunn, R. J.; Ivetac, A., Fragment-based discovery of MRTX1719, a synthetic lethal inhibitor of the PRMT5• MTA complex for the treatment of MTAP-deleted cancers. Journal of Medicinal Chemistry 2022, 65 (3), 1749-1766.

8:Engstrom, L. D.; Aranda, R.; Waters, L.; Moya, K.; Bowcut, V.; Vegar, L.; Trinh, D.; Hebbert, A.; Smith, C. R.; Kulyk, S., MRTX1719 is an MTA-cooperative PRMT5 inhibitor that exhibits synthetic lethality in preclinical models and patients with MTAP-deleted cancer. Cancer discovery 2023, 13 (11), 2412-2431.

9:Cottrell, K. M.; Briggs, K. J.; Whittington, D. A.; Jahic, H.; Ali, J. A.; Davis, C. B.; Gong, S.; Gotur, D.; Gu, L.; McCarren, P., Discovery of TNG908: A Selective, Brain Penetrant, MTA-Cooperative PRMT5 Inhibitor That Is Synthetically Lethal with MTAP-Deleted Cancers. Journal of Medicinal Chemistry 2024, 67 (8), 6064-6080.

10: Colquhoun, D. Why the Schild method is better than Schild realised. Trends Pharmacol. Sci. 28, 608 614, doi:10.1016/j.tips.2007.09.011 (2007).

11: Morrison, J. Kinetics of the reversible inhibition of enzyme-catalysed reactions by tight-binding inhibitors. Biochimica et Biophysica Acta (BBA)-Enzymology 185, 269-286 (1969).

Uncropped Western blots for Figure S1:

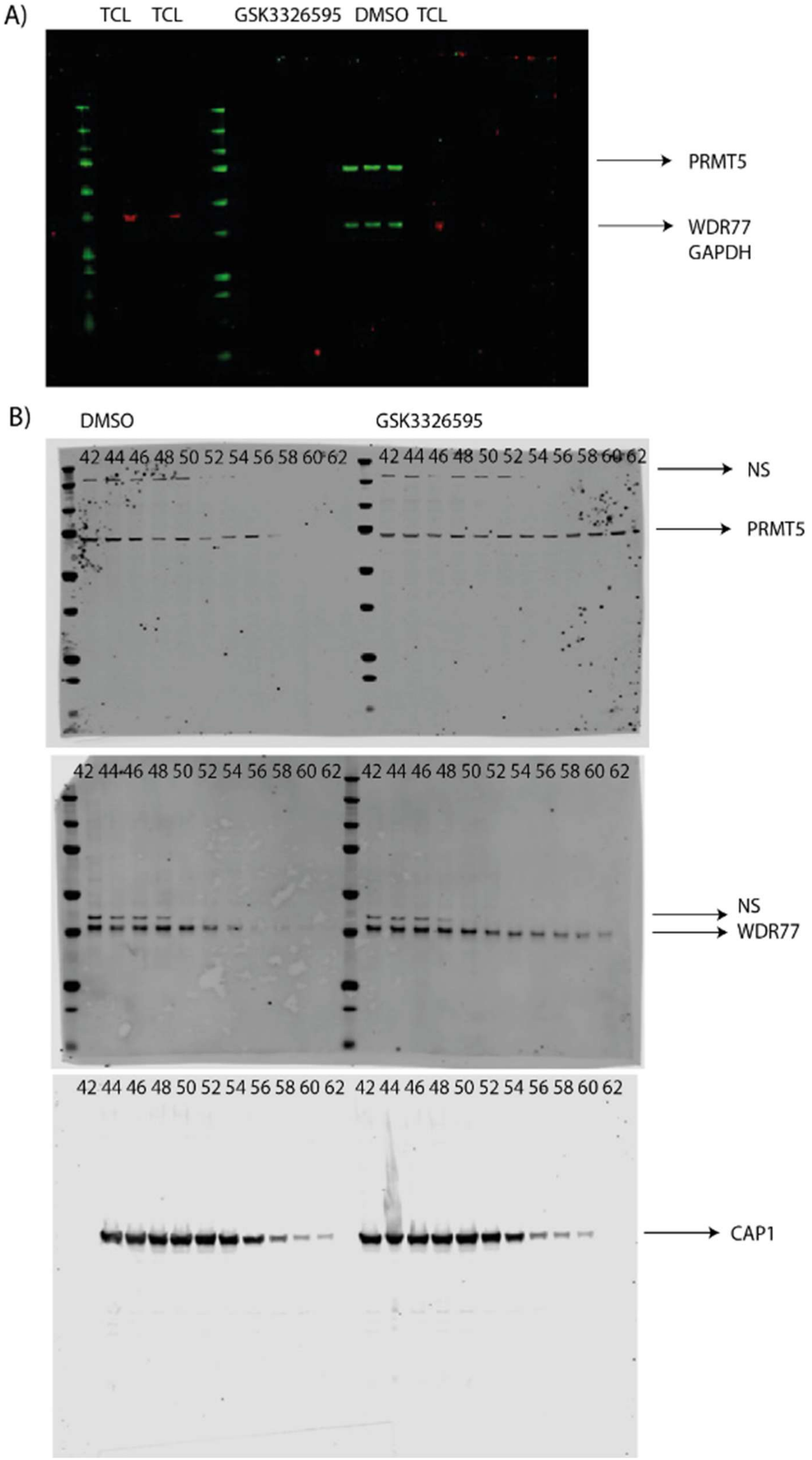

Supplement: Supplementary file 1 — Supplementary Information [file 41467_2025_65558_MOESM1_ESM.pdf]
