# Supplementary material for: A BRET biosensor for measuring uncompetitive engagement of PRMT5 complexes in cells
Source: Nat Commun. 2025 Dec 3;16:10129. doi: 10.1038/s41467-025-65558-6 (PMC12675575; doi:10.1038/s41467-025-65558-6)
Supplement: Supplementary file 2 — Reporting Summary [file 41467_2025_65558_MOESM2_ESM.pdf]

nature portfolio

Corresponding author(s): Kilian VM Huber, Matthew Robers, Peter Tonge

Last updated by author(s): Oct 10, 2025

Reporting Summary

Nature Portfolio wishes to improve the reproducibility of the work that we publish. This form provides structure for consistency and transparency in reporting. For further information on Nature Portfolio policies, see our Editorial Policies and the Editorial Policy Checklist.

Statistics

For all statistical analyses, confirm that the following items are present in the figure legend, table legend, main text, or Methods section.

n/a	Confirmed	
☐	☒	The exact sample size (n) for each experimental group/condition, given as a discrete number and unit of measurement
☐	☒	A statement on whether measurements were taken from distinct samples or whether the same sample was measured repeatedly
☒	☐	The statistical test(s) used AND whether they are one- or two-sided<br>*Only common tests should be described solely by name; describe more complex techniques in the Methods section.*
☒	☐	A description of all covariates tested
☒	☐	A description of any assumptions or corrections, such as tests of normality and adjustment for multiple comparisons
☐	☒	A full description of the statistical parameters including central tendency (e.g. means) or other basic estimates (e.g. regression coefficient) AND variation (e.g. standard deviation) or associated estimates of uncertainty (e.g. confidence intervals)
☒	☐	For null hypothesis testing, the test statistic (e.g. F, t, r) with confidence intervals, effect sizes, degrees of freedom and P value noted<br>*Give P values as exact values whenever suitable.*
☒	☐	For Bayesian analysis, information on the choice of priors and Markov chain Monte Carlo settings
☒	☐	For hierarchical and complex designs, identification of the appropriate level for tests and full reporting of outcomes
☒	☐	Estimates of effect sizes (e.g. Cohen's d, Pearson's r), indicating how they were calculated

Our web collection on statistics for biologists contains articles on many of the points above.

Software and code

Policy information about availability of computer code

Data collection	Xcalibur v1.4 (Thermo), Proteome Discoverer v2.1 (Thermo Fisher), GraphPad Prism v8, ChemBioDraw Ultra v19.0 (Perkin Elmer) , MestreNova v14.2.1 (Mestre Lab Research), Perseus 1.6.2.1 (Max-Planck-Institute of Biochemistry), MaxQuant 1.6.5 (Max-Planck-Institute of Biochemistry)
Data analysis	The data for 2D CETSA was analysed using an in-house MATLAB script that represents the temperature-abundance matrix for each protein as a surface and fits a surface equation. The amplitude of the thermal stabilization is calculated using the volume of the surface, and plotted against the goodness-of-fit to the surface equation. Code available in: Stefaniak J. (2019). Cellular target engagement and selectivity profiling of small molecules for mechanism-of-action studies (PhD thesis). University of Oxford<br>Binding data were analyzed by fitting data to dose response equations in Graphpad Prism using non-linear regression. The goodness of fit is assessed by calculating 95% confidence intervals around the fitted curve, representing the range within which the true curve is likely to lie.

For manuscripts utilizing custom algorithms or software that are central to the research but not yet described in published literature, software must be made available to editors and reviewers. We strongly encourage code deposition in a community repository (e.g. GitHub). See the Nature Portfolio guidelines for submitting code & software for further information.

Data

Policy information about availability of data

All manuscripts must include a data availability statement. This statement should provide the following information, where applicable:

- Accession codes, unique identifiers, or web links for publicly available datasets
- A description of any restrictions on data availability
- For clinical datasets or third party data, please ensure that the statement adheres to our policy

The proteomics data generated in this study have been deposited in the PRIDE database under accession code PXD028138 [http://proteomecentral.proteomexchange.org/cgi/GetDataset?ID=PXD028138].

Research involving human participants, their data, or biological material

Policy information about studies with human participants or human data. See also policy information about sex, gender (identity/presentation), and sexual orientation and race, ethnicity and racism.

Reporting on sex and gender	NA
Reporting on race, ethnicity, or other socially relevant groupings	NA
Population characteristics	NA
Recruitment	NA
Ethics oversight	NA

Note that full information on the approval of the study protocol must also be provided in the manuscript.

Field-specific reporting

Please select the one below that is the best fit for your research. If you are not sure, read the appropriate sections before making your selection.

☒ Life sciences ☐ Behavioural & social sciences ☐ Ecological, evolutionary & environmental sciences

For a reference copy of the document with all sections, see nature.com/documents/nr-reporting-summary-flat.pdf

Life sciences study design

All studies must disclose on these points even when the disclosure is negative.

Sample size	Sample size was at least n=3 which is sufficient to provide standard deviations
Data exclusions	Only for curve fitting purposes, in some cases a single outlier data point was excluded
Replication	To ensure reproducibility, a minimum of 3 experimental replicates was performed
Randomization	*Describe how samples/organisms/participants were allocated into experimental groups. If allocation was not random, describe how covariates were controlled OR if this is not relevant to your study, explain why.*
Blinding	*Describe whether the investigators were blinded to group allocation during data collection and/or analysis. If blinding was not possible, describe why OR explain why blinding was not relevant to your study.*

Reporting for specific materials, systems and methods

We require information from authors about some types of materials, experimental systems and methods used in many studies. Here, indicate whether each material, system or method listed is relevant to your study. If you are not sure if a list item applies to your research, read the appropriate section before selecting a response.

Materials & experimental systems

n/a	Involved in the study
☐	☒ Antibodies
☐	☒ Eukaryotic cell lines
☒	☐ Palaeontology and archaeology
☒	☐ Animals and other organisms
☒	☐ Clinical data
☒	☐ Dual use research of concern
☒	☐ Plants

Methods

n/a	Involved in the study
☒	☐ ChIP-seq
☒	☐ Flow cytometry
☒	☐ MRI-based neuroimaging

Antibodies

Antibodies used

Anti-MEP50 (Cell Signaling Cat#2823), Anti-PRMT5 (EPR5772) (Abcam, Cat#ab-109451), Anti-GAPDH (G-9) (Santa Cruz, Cat#s-365062), Anti-CAP1 (D2K3J) (Cell Signaling, Cat#47055)

Validation

Anti-MEP50 (Cell Signaling Cat#2823): reactivity human, sensitivity endogenous, key applications WB-Western Blotting IP-Immunoprecipitation IF-Immunofluorescence (https://www.cellsignal.com/products/primary-antibodies/mep50-antibody/2823?srsltid=AfmBOoqswiAOou3MkjfqDxKbUlpbFhTJMvaPb2H1szOKEEltZyYgxCVR). Anti-PRMT5 (EPR5772) (Abcam, Cat#ab-109451)Rabbit Recombinant Monoclonal PRMT5 antibody, Suitable for IHC-P, IP, WB, ICC/IF, Flow: Cyt (Intra) and reacts with Mouse, Human, Rat samples, https://www.abcam.com/en-us/products/primary-antibodies/prmt5-antibody-epr5772-ab109451?srsltid=AfmBOoqBIHY8lK4VJwjQJFbbSd6nsphWwuZYoFhChqEVeoXVsxPDzVQg. Anti-GAPDH (G-9) (Santa Cruz, Cat#s-365062):recommended for detection of GAPDH and GAPDH-2 of mouse, rat and human origin by WB, IP, IF, IHC(P) and ELISA https://www.scbt.com/p/gapdh-antibody-g-9?srsltid=AfmBOoqNEXQeD5t3Tvi6JVil6BKEiH6VL5RNZPFx8pw-WUqjhg-sErM_. Anti-CAP1 (D2K3J) (Cell Signaling, Cat#47055): reactivity human mouse rat, sensitivity endogenous, key applications WB-Western Blotting, https://www.cellsignal.com/products/primary-antibodies/cap1-d2k3j-rabbit-mab/47055?srsltid=AfmBOor8_0NAKGmWsHOFknOxWCqVOElt82EgL7R9BRPfaboN1_SC8sc0

Eukaryotic cell lines

Policy information about cell lines and Sex and Gender in Research

Cell line source(s)

HEK293 (ATCC, CRL-1573.3), KMS11 (Laboratory of Martin Kaiser, ICR London), HCT-116 (Horizon, R02-033, PAR-034)

Authentication

ATCC and Horizon authenticated cell lines, KMS11 were not authenticated.

Mycoplasma contamination

All cell lines were negative for mycoplasma

Commonly misidentified lines
(See ICLAC register)

N/A

Plants

Seed stocks

Report on the source of all seed stocks or other plant material used. If applicable, state the seed stock centre and catalogue number. If plant specimens were collected from the field, describe the collection location, date and sampling procedures.

Novel plant genotypes

Describe the methods by which all novel plant genotypes were produced. This includes those generated by transgenic approaches, gene editing, chemical/radiation-based mutagenesis and hybridization. For transgenic lines, describe the transformation method, the number of independent lines analyzed and the generation upon which experiments were performed. For gene-edited lines, describe the editor used, the endogenous sequence targeted for editing, the targeting guide RNA sequence (if applicable) and how the editor was applied.

Authentication

Describe any authentication procedures for each seed stock used or novel genotype generated. Describe any experiments used to assess the effect of a mutation and, where applicable, how potential secondary effects (e.g. second site T-DNA insertions, mosiacism, off-target gene editing) were examined.
